# Adherence, Sexual Behavior and Sexually Transmitted Infections in a New Zealand Prospective PrEP Cohort: 12 Months Follow-up and Ethnic Disparities

**DOI:** 10.1007/s10461-022-03617-5

**Published:** 2022-02-15

**Authors:** Peter J. W. Saxton, Sunita Azariah, Alana Cavadino, Rose F. Forster, Renee Jenkins, Suzanne F. Werder, Kim Southey, Joseph G. Rich

**Affiliations:** 1 School of Population Health, University of Auckland, 28 Park Ave, Auckland, 1023 New Zealand; 2 Auckland Sexual Health Regional Service, Auckland District Health Board, Auckland, New Zealand; 3 Department of Obstetrics and Gynaecology, University of Auckland, Auckland, New Zealand; 4 Te Whāriki Takapou, Waikato, New Zealand; 5 New Zealand AIDS Foundation, Auckland, New Zealand

**Keywords:** Race, Implementation, HIV, PrEP, Adherence

## Abstract

Inequities in pre-exposure prophylaxis (PrEP) experiences will impede HIV epidemic elimination among gay and bisexual men (GBM). Ethnicity is a strong marker of inequity in the United States, but evidence from other countries is lacking. We investigated experiences on-PrEP to 12 months follow-up in a prospective cohort of 150 GBM in Auckland, New Zealand with an equity quota of 50% non-Europeans. Retention at 12 months was 85.9%, lower among Māori/Pacific (75.6%) than non-Māori/Pacific participants (90.1%). Missed pills increased over time and were higher among Māori/Pacific. PrEP breaks increased, by 12 months 35.7% of Māori/Pacific and 15.7% of non-Māori/Pacific participants had done so. Condomless receptive anal intercourse partners were stable over time. STIs were common but chlamydia declined; 12-month incidence was 8.7% for syphilis, 36.0% gonorrhoea, 46.0% chlamydia, 44.7% rectal STI, 64.0% any STI. Structural interventions and delivery innovations are needed to ensure ethnic minority GBM gain equal benefit from PrEP.

*Clinical trial number* ACTRN12616001387415.

**Supplementary Information:**

The online version contains supplementary material available at 10.1007/s10461-022-03617-5.

## Introduction

Gay, bisexual and other men who have sex with men (GBM) are at elevated risk of HIV [1, 2] and in many countries comprise the largest share of new diagnoses [3–7]. Pre-exposure prophylaxis (PrEP) protects against HIV if taken as prescribed [8–10], with at least 86% risk-reduction in real world trials [11, 12]. PrEP is now an essential component of combination HIV prevention for GBM including condoms, HIV testing and viral suppression of people living with HIV [3, 13]. Modelling suggests that high PrEP uptake can help eliminate HIV transmission at the population level [14–16] and ecological data support this [17, 18].

For PrEP to fulfill its potential, HIV negative GBM must attend clinical services; consequently, implementation science must help us understand barriers to PrEP engagement [19–24]. Behavioral risk compensation also interests researchers [25, 26], including risks of non-HIV sexually transmitted infections (STI) [27]. Prospective open-label cohorts (“demonstration projects”) have identified heterogenous outcomes associated with PrEP use [28–38]. For example, reviews have shown that adherence can be associated with lower perceived stigma, greater empowerment, experiences of fewer side-effects, positive provider interactions, lower cost and less segmented health systems [39–41], that PrEP is associated with changes in condomless anal intercourse [42, 43] and that STI incidence remains high in GBM taking PrEP [27, 44, 45].

A particular concern is the effectiveness of PrEP for ethnic minority GBM. Ethnic minority GBM may have greater HIV prevention needs [46, 47], similar interest in and uptake of PrEP to ethnic majority GBM [48], yet report poorer PrEP outcomes post-initiation [49–51]. US studies have identified the important role that social determinants of health play in generating these disparities, including structural racism, that place ethnic minority GBM at a disadvantage when navigating health systems [52–54]. However, little is known about the experience of ethnic minority GBM on PrEP outside the US.

New Zealand offers a unique setting to examine PrEP delivery with an equity lens. Auckland is an ethnically superdiverse city [55] with publicly-funded sexual health clinics. In 2018, PrEP was fully funded for indviduals at elevated HIV risk [56], estimated at 17.9% of HIV negative GBM [57]. At the same time, evidence shows that both Māori and Pacific people, experience substantial health inequities in New Zealand [58, 59] that are driven by social determinants, racism and unconscious bias [60]. Māori GBM have a similar HIV prevalence to non-Māori GBM [61, 62], but diagnosis occurs later [63] and condom use with casual partners is lower [64, 65]. Therefore, PrEP will benefit ethnic minority GBM, but poor implementation could inadvertently widen disparities [56].

We aimed to investigate trends in PrEP adherence, sexual behavior and STIs over 12 months follow-up in a prospective cohort of PrEP users in New Zealand. We also examined whether the experiences of ethnic minority GBM were similar or diverged to other GBM.

## Methods

### Design, Enrolment, Equity Quota

The NZPrEP study was a prospective open-label, single-arm treatment evaluation study (“demonstration project”). The aim was to inform PrEP implementation in Auckland, New Zealand. The protocol [66] and baseline findings [67] are reported elsewhere. Briefly, GBM at elevated risk of HIV acquisition, eligible for publicly-funded healthcare and residing in Auckland were enrolled at one of four free sexual health clinics from February 2017 and the final visit was completed February 2019. Participants were recruited from community and clinic settings and could self-refer or be referred by a doctor or nurse. Written informed consent was obtained. We followed participants for a maximum of 48 weeks. The sample was capped at 150 participants with an equity quota of 75 European and 75 non-Europeans. This gave us the following statistical power: (1) assuming 80% retention at 48 weeks in Europeans, we had 85% power to detect a 20% lower retention rate in non-Europeans; (2) assuming 80% retention at 48 weeks in Europeans, we had 72% power to detect a 20% lower retention rate in Māori, if Māori comprise 20% of the entry sample; (3) assuming 10% reported elevated-risk behavior (10 + receptive anal intercourse partners in the preceding three months) at baseline, we had 81% power to detect an increase to 21% at 48 weeks if 120 participants were retained, based on the UK PROUD trial experience [11]. The study received ethics approval from the New Zealand Health and Disability Ethics Committee (#16/NTA/112).

### Procedures

Participants attended three-monthly clinic visits for testing, PrEP prescriptions and assessment of risk and adverse events. We combined the month 1 and 3 visits into visit 1, therefore we report four visits over 12 months. Prescriptions could be dispensed at one of two participating community pharmacies. We recommended daily PrEP and did not offer an event-based dosing option (“2-1-1”) [66]. If a participant wished to stop PrEP, they were advised to continue daily dosing for 28 days after the last risk episode. Participants wishing to re-start were advised to receive a negative HIV test then use condoms or abstain for seven days to allow protective levels of drug to build up in rectal tissue. Study medication was supplied by Gilead (Truvada™). Within three days of a visit, we invited participants to self-complete an anonymous follow-up behavioral survey online using SurveyMonkey (San Mateo, CA, USA). Reminders were issued by email and text message. Survey data were held securely at the University of Auckland, separate from clinic records. Data linkage was via a unique study number. No financial incentives were offered to complete the survey.

### Measures

We used the baseline survey socio-demographic data where participants could claim multiple ethnicities. The follow-up surveys included a wide range of items canvassing adherence, sexual and drug use behaviors and attitudes to taking PrEP. Incident STIs at follow-up (chlamydia, gonorrhoea, syphilis, non-specific urethritis (NSU), proctitis, genital herpes and genital warts) were laboratory or clinician confirmed. We assessed PrEP adherence by self-reported missed doses over: last seven days; last 30 days; last 3 months. We also asked participants their self-efficacy of taking the pills, frequency and severity of side effects, reasons for missing doses, periodic breaks from taking pills, length of and reason for breaks, and missed appointments. Sexual behavior in the prior three months recorded the following: number of male sexual partners (“Men”); number of male partners had condomless anal intercourse with (“MenAICL”); number of male partners had receptive condomless anal intercourse with (“MenAICLR”). Frequency of substance use before or during sex in the previous three months was reported on a five point scale (never, some of the time, half the time, most of the time, always) and included amyl nitrate, cannabis, GHB, ecstasy, amphetamine, methamphetamine, cocaine, ketamine, LSD, mephedrone, “other” and alcohol (separately asked). Attitudes to PrEP, for example communication to sexual partners and concerns about the future, were asked on a five point scale (e.g. strongly agree, agree, neither agree nor disagree, disagree, strongly disagree).

### Data Analysis

Missed PrEP doses and sexual partnering were summarised by mean, standard deviation (SD), median and inter-quartile range (IQR). We also report the overall proportion who reported “any” such behaviors, and for sexual behavior, the proportion who reported 10 or more partners. Substance use was dichotomised into “heavy use” (most of the time or always) or “none or moderate use”, and “chemsex” was defined as using methamphetamine, GHB or mephedrone before sex. Attitudes were dichotomised into “strongly agree or agree” versus other responses.

We allocated participants to a binary “Māori/Pacific” or “non-Māori/Pacific” ethnicity (including those identifying as European, Asian, Latin-American or Other but not as Māori or Pacific). This equity measure varied from the initial aim, but reflects local patterns of ethnic inequities and increased the equity sub-sample from n = 32 (21.3% of 150) to 42 (28%). We compared the entry characteristics of participants retained and not retained at 12 months using chi-squared tests and Fisher’s exact tests where appropriate. We examined time-to-study-exit using Kaplan–Meier survival estimates. Next, we investigated trends in adherence, behaviors and STIs using multilevel mixed-effects Poisson regression for count data and multilevel mixed-effects logistic regression for binary variables; each approach takes account of loss-to-follow-up and the reported p-value compares baseline or visit 1 with visit 4 (12 months), along with odds ratios (OR) and relative risks (RR) with 95% confidence intervals (CI). We describe experiences at each visit, the average per visit across 12 months and the overall proportion over 12 months. We used nonparametric tests to examine changes over time in attitudes to PrEP. Finally, we compared outcomes between Māori/Pacific and non-Māori/Pacific using the same technique described above for continuous and binary variables but with both time and ethnicity entered as covariates. The denominator for analyses of clinically-verified STIs was all participants; for analyses of behaviors it was completed surveys. All statistical analyses were conducted using Stata version 14.0 (StataCorp, College Station, TX, USA) with an alpha of 5% (p < 0.05).

## Results

Of the 150 participants enrolled, eight (one Maōri/Pacific, seven non-Māori/Pacific) left Auckland rendering them ineligible. Of the remaining 142, 122 (85.9%) were retained at 12 months. Of the 20 not retained, six completed an early exit survey, five exited because they stopped PrEP (four decided to stop and one said their doctor recommended stopping) and one obtained PrEP elsewhere. Reasons for stopping included: difficulties taking the pill everyday or arranging convenient clinic appointments (2); using condoms or starting a relationship with an HIV negative partner (2); not wanting to be on PrEP (2) (multiple reasons permitted). Table 1 shows the retention rate at 12 months according to participants’ baseline characteristics. Māori/Pacific participants were less likely to be retained (75.6%) compared to non-Māori/Pacific (90.1%) (chi-square = 5.06, p = 0.024). No other significant differences were identified. Supplementary Figure S1 displays the time-to-exit of the 142 participants by ethnicity group. Overall retention was 98.6% at 3 months, 91.6% at 6 months, 88.7% at 9 months and 85.9% at 12 months.

**Table 1:** Retention at 12 months by baseline characteristics of NZPrEP participants

Characteristic	Retained		Not retained		Test statistic (df)	P value (chi-square)
	n	%	n	%		
Overall	122	85.9	20	14.1		
Site					1.09 (2)	0.297
Central	103	87.3	15	12.7		
Other	19	79.2	5	20.8		
Age					0.24 (2)	0.624
18–29	48	84.2	9	15.8		
30 +	68	87.2	10	12.8		
Ethnicity					5.06 (2)	**0.024**
Māori/Pacific	31	75.6	10	24.4		
Non-Māori/Pacific	91	90.1	10	9.9		
Sexual identity					n/a	0.187^a^
Gay	114	87.0	17	13.0		
Bisexual or other	8	72.7	3	27.3		
Highest education					0.23 (2)	0.584
Less than tertiary degree	53	84.1	10	15.9		
Tertiary degree	69	87.3	10	12.7		
Referral status					2.47 (2)	0.116
Participant	83	89.3	10	10.8		
Doctor or nurse	39	79.6	10	20.4		
STI diagnosed < 12 m					2.23 (2)	0.136
No	39	79.6	10	20.4		
Yes	80	88.9	10	11.1		
Prevalent STI					n/a	0.783^a^
None	92	85.2	16	14.8		
Any	30	88.2	4	11.8		
PEP use ever					n/a	0.765^a^
No	96	85.7	16	14.3		
Yes	25	89.3	3	10.7		
10 or more condomless receptive anal sex partners					n/a	0.418^a^
No	111	86.7	17	13.3		
Yes	11	78.6	3	21.4		
Group sex					0.18 (2)	0.672
No	67	84.8	12	15.2		
Yes	56	88.9	7	11.1		
Alcohol use before sex < 3 m					n/a	0.739^a^
None or moderate	104	85.3	18	14.8		
Heavy	18	90.0	2	10.0		
Chemsex < 3 m					n/a	1.000^a^
No	103	85.8	17	14.2		
Yes	18	85.7	3	14.3		
Erection dysfunction medication < 3 m					0.05 (2)	0.831
No	75	86.2	12	13.8		
Yes	45	84.9	8	15.1		
Motivated to take PrEP?					n/a	0.259^a^
Completely	116	86.6	18	13.4		
Somewhat or not	5	71.4	2	28.6		
Acceptability of PrEP?					n/a	1.000^a^
Completely	85	85.9	14	14.1		
Somewhat or not	36	85.7	6	14.3		
Likelihood of remaining in study?					n/a	0.231^a^
Extremely	109	87.2	16	12.8		
Somewhat or less	11	73.3	4	26.7		

Bold denotes statistically significant

*df* degrees of freedom, *n/a* not applicable, *PEP* post-exposure prophylaxis

^a^Fisher’s exact test

### 12 Months Follow-up: All Participants

Engagement with the three-monthly follow-up surveys was 89.3% at visit 1 and 70.7% at visit 4 (Table 2). On average, 11.4% reported missing a scheduled appointment at any one visit; overall 22.7% reported missing an appointment at least once over 12 months.

**Table 2:** PrEP adherence, study engagement, sexual behavior and drug use among NZPrEP survey respondents to 12 months follow-up

Variable	Baseline		Visit 1 (months 0–3) n = 134		Visit 2 (months 3–6) n = 117		Visit 3 (months 6–9) n = 117		Visit 4 (months 9–12) n = 106		Average per visit	Overall	OR, RR (95% CI)	P value comparing visit 4 vs 1 or baseline
	n	%	n	%	n	%	n	%	n	%	%	%		
Survey engagement	150	*100.0*	134	*89.3*	117	*78.0*	117	*78.0*	106	*70.7*	n/a	n/a		
Missed scheduled appointment			16	*12.0*	11	*9.5*	14	*12.2*	12	*11.8*	*11.4*	*22.7*	1.05 (0.41–2.71)	0.912^a^
*PrEP adherence*														
Missed any pills < 7 days			35	*26.1*	29	*24.8*	38	*32.5*	24	*22.6*	n/a	n/a	0.83 (0.42–1.65)	0.604^a^
Missed 4 or more < 7 days			1	*0.8*	2	*1.7*	2	*1.7*	5	*4.7*	n/a	n/a		Not tested
Missed 4 or more < 30 days			11	*8.2*	10	*8.6*	15	*12.8*	19	*17.9*	n/a	n/a	**5.63 (1.71–18.56)**	**0.005**^a^
No. missed pills < 3 months; mean (SD)			2.97	(8.21)	3.63	(9.47)	3.72	(4.65)	5.81	(15.11)	n/a	n/a	**2.90 (2.49–3.38)**	** < 0.001**^a^
Self-rated “taking the pills is easy” (strongly agree)			74	*54.8*	70	*59.8*	70	*59.8*	59	*55.7*	*57.5*	n/a	0.90 (0.46–1.76)	0.751^a^
Self-rated “how well did you do at taking the pills?” (extremely well)			83	*61.9*	77	*66.4*	74	*63.3*	67	*63.2*	*63.6*	n/a	1.0 (0.51–1.97)	0.994^a^
*PrEP breaks and side effects*														
Had a break from pills			5	*3.8*	7	*6.0*	13	*11.1*	15	*14.2*	*8.5*	*21.3*	**6.27 (1.80–21.81)**	**0.004**^a^
Length of break was more than 2 weeks			2	*33.3*	3	*42.9*	4	*30.8*	6	*40.0*	*36.6*	n/a	1.26 (0.31–51.02)	0.903^a^
Experienced side effects			46	*34.3*	24	*20.5*	18	*15.5*	12	*11.3*	*21.1*	*35.3*	**0.04 (0.01–0.15)**	** < 0.001**^a^
Severity of side effects; mean (SD)§			25.2	(19.7)	21.3	(18.8)	21.5	(23.7)	27.3	(12.3)	n/a	n/a	**0.69 (0.58–0.81)**	** < 0.001**^b^
*Sexual behaviors*														
Men; mean (SD)	15.2	(12.4)	13.4	(12.7)	12.1	(12.4)	11.4	(12.6)	11.1	(13.20	n/a	n/a	**0.71 (0.66–0.77)**	** < 0.001**^b^
10 or more Men	92	*61.3*	73	*55.7*	53	*45.7*	54	*47.0*	46	*45.5*	*51.9*	n/a	**0.27 (0.13–0.57)**	**0.001**^a^
Any MenAICL	146	*97.3*	123	*93.2*	114	*98.3*	112	*97.4*	99	*98.0*	*96.7*	*90.0*	1.23 (0.17–8.70)	0.833^a^
MenAICL; mean (SD)	6.5	(7.0)	7.3	(9.7)	7.3	(10.0)	6.8	(10.2)	6.9	(10.7)	n/a	n/a	1.05 (0.95–1.16)	0.372^b^
10 + MenAICL	34	*22.7*	33	*25.0*	22	*19.0*	25	*21.7*	18	*17.8*	*21.5*	n/a	0.89 (0.74–1.06)	0.196^a^
Any MenAICLR	141	*96.6*	114	*86.4*	100	*86.2*	105	*91.3*	85	*84.2*	*89.3*	*86.7*	**0.04 (0.01–0.20)**	**0.001**^a^
MenAICLR; mean, (SD)	4.9	(5.7)	5.0	(7.7)	5.4	(9.3)	5.3	(9.6)	5.3	(10.3)	n/a	n/a	1.10 (0.98–1.24)	0.098^b^
10 or more MenAICLR	15	*10.0*	18	*13.6*	13	*11.2*	19	*16.5*	14	*13.9*	*12.9*	n/a	2.0 (0.68–5.85)	0.207^a^
*Substance use*														
Alcohol use before sex < 3 m (heavy)	20	*13.3*	15	*11.2*	12	*10.3*	16	*13.9*	11	*10.7*	*12.0*	*17.3*	0.55 (0.16–1.91)	0.343^a^
Drug use before sex < 3 m (heavy)	15	*10.1*	7	*5.2*	7	*6.1*	6	*5.2*	3	*2.9*	*6.2*	*10.0*	0.17 (0.03–1.16)	0.071^a^
Methamphetamine use before sex < 3 m (any)	12	*8.1*	9	*6.7*	6	*5.2*	5	*4.4*	4	*3.9*	*5.8*	*8.7*	0.30 (0.04–2.08)	0.223^a^
Chemsex < 3 m	23	*15.4*	20	*14.9*	16	*13.9*	16	*13.9*	15	*14.6*	*14.6*	*19.3*	1.12 (0.33–3.80)	0.850^a^
Injected drugs < 3 m			2	*1.5*	2	*1.7*	3	*2.6*	1	*1.0*	*1.7*	*4.0*		Not tested

*SD* standard deviation

*n/a* not applicable

Bold denotes statistically significant

Italics represent proportions

Overall cumulative % may be lower than a visit % for some outcomes due to survey attrition. Men = No. male partners. MenAICL = No. male partners had condomless anal intercourse with. MenAICLR = No. male partners had condomless receptive anal intercourse with

P values compare baseline or visit 1 with visit 4 (12 months) along with the OR or RR and 95% CI using the following statistical tests:

^a^For categorical data, multilevel fixed effects logistic regression

^b^For continuous data (counts), multilevel fixed effects Poisson regression

^c^Of those experiencing side-effects

Adherence was high but declined over time. In the seven days prior to a visit, on average a quarter of participants missed one or more PrEP pills (Table 2), however few (generally < 2%) missed four or more, which would indicate reduced effectiveness. In the 30 days prior to a visit, there was an increase in the proportion missing four or more doses at visit 4 (OR 5.63, 95% CI 1.71–18.56) and in the number of missed doses (RR 2.33, 95% CI 1.90–2.85) compared to visit 1, although the median number of missed doses was low at one. In the three months prior to visit, there was an increase in the mean (2.97–5.81) and in the median number of missed doses (1–2, RR 2.90, 95% CI 2.49–3.38) between visits 1 and 4. On average 57.5% strongly agreed that “taking the pills is easy” and 63.6% believed they did “extremely well” at taking the pills; neither measure changing over time (Table 2). The most common reasons for missing pills were “I forgot” (50.9%), “I was away from home” (44.5%), “my daily routine changed” (34.1%) and “I was too busy” (10.7%) (Supplementary Table S1).

**Fig. 1:**
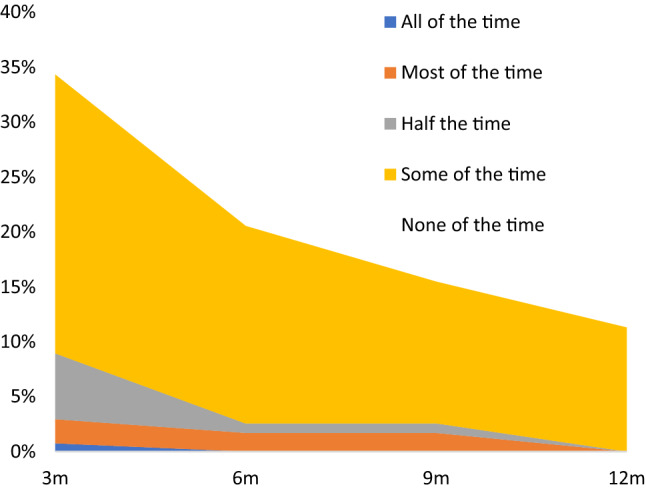
Proportion of NZPrEP participants experiencing side-effects in the previous 3 months, over 12 months follow-up

An increasing proportion took a PrEP break in the three months between study visits, ranging from 3.8% at visit 1 to 14.2% at visit 4 (OR 6.27, 95% CI 1.80–21.81). Most breaks lasted 3–7 days (34.2%), followed by 8–14 days (24.4%) and more than two weeks (22.0%). The most common reasons for taking a break in the previous three months were “I was not having sex” (30.0%), “I didn’t feel at risk of HIV (17.5%), “I was worried about interactions with other drugs” (17.5%), “I was doing event-based dosing” (15.0%), “I was using condoms” (12.5%) and “I was not feeling well” (10.0%) (Supplementary Table S2). Overall 35.3% of participants reported experiencing side-effects at least once over 12 months, with most experiencing this in the first three months (34.3%) declining to 11.3% by visit 4 (OR 0.04, 95% CI 0.01–0.15) (Table 2 and Fig. 1). Among participants reporting side-effects, the severity changed over time (declining from a mean 25.2/100 at visit 1 then rising again to 27.3/100 at visit 4) (RR 0.69, 95% CI 0.58–0.81, Table 2). Despite imperfect adherence, no participant acquired HIV during study follow-up.

Participants reported fewer male sexual partners over time [median (IQR) for baseline = 10.5 (7–20), visit 4 = 6 (4–12); RR 0.71, 95% CI 0.66–0.77] [for mean (SD), see Table 2]. However, the number of condomless receptive anal intercourse partners was steady [median (IQR) for baseline = 3 (2–7) vs visit 4 = 2 (1–5); RR 1.10, 95% CI 0.98–1.24] [for mean, (SD) see Table 2]. The proportion reporting 10 or more such partners (baseline = 10.0% vs visit 4 = 13.9%, OR 2.0, 95% CI 0.68–5.85) did not change significantly over follow-up.

**Fig. 2:**
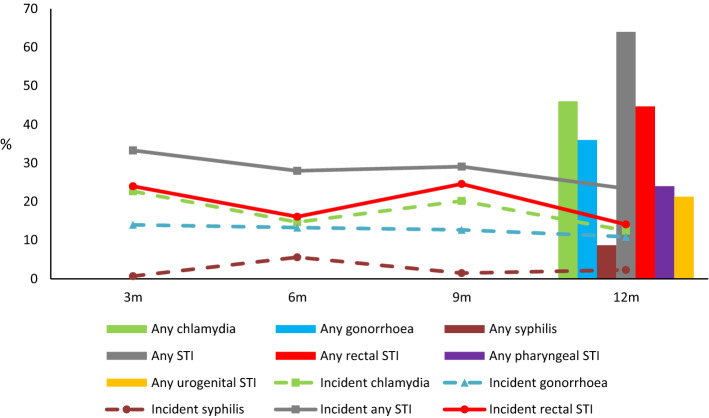
3 month incidence and any sexually transmitted infection among NZPrEP participants to 12 months follow-up

There were 195 incidences of STI diagnosed over 133.25 years of follow-up (Table 3). The standardised incidence of any chlamydia, gonorrhoea or syphilis was 137.3/100py. STI incidence remained high but there were declines in chlamydia (OR 0.44, 95% CI 0.22–0.97) and the composite any STI (OR 0.56, 95% CI 0.32–0.99) at visit 4 compared to visit 1 (Table 3 and Fig. 2). On average, 28.7% of participants were diagnosed with one or more STIs per visit, including 1 in 5 (19.8%) with a rectal STI. Overall, the 12-month incidence of diagnosed infection was: chlamydia (46.0%); gonorrhoea (36.0%); syphilis (8.7%); any STI (64.0%); rectal STI (44.7%); pharyngeal STI (24.0%) and urogenital STI (21.3%).

**Table 3:** Sexually transmitted infections among NZPrEP participants to 12 months follow-up

Diagnosis	Visit 1 n = 150		Visit 2 n = 143		Visit 3 n = 134		Visit 4 n = 128		Average per visit	Overall	OR (95% CI)	P value comparing visit 4 vs 1^a^	Incidences	Incidence per 100py (95% CI)
	n	%	n	%	n	%	n	%	%	%				
Chlamydia	34	*22.7*	21	*14.7*	27	*20.2*	16	*12.5*	*17.7*	*46.0*	**0.44 (0.22–0.97)**	**0.019**	98	73.6 (60.0–89.2)^b^
Gonorrhoea	21	*14.0*	19	*13.3*	17	*12.7*	14	*10.9*	*12.8*	*36.0*	0.72 (0.33–1.58)	0.416	71	53.3 (41.9–66.8)^b^
Syphilis	1	*0.7*	8	*5.6*	2	*1.5*	3	*2.3*	*2.5*	*8.7*	3.67 (0.37–36.66)	0.268	14	10.5 (6.0–17.2)
Chlamydia, gonorrhoea, syphilis	46	*30.7*	39	*27.3*	38	*28.4*	27	*21.1*	*27.0*	*61.3*	**0.55 (0.31–0.99)**	**0.049**	183	137.3 (118.5–158.4)^b^
Any STI	50	*33.3*	40	*28.0*	39	*29.1*	30	*23.4*	*28.7*	*64.0*	**0.56 (0.32–0.99)**	**0.048**	195	146.3 (126.9–168.0)^c^
Any rectal STI	36	*24.0*	23	*16.1*	33	*24.6*	18	*14.1*	*19.8*	*44.7*	**0.40 (0.20–0.82)**	**0.013**	130	97.6 (81.8–115.5)^d^
Any pharyngeal STI	9	*6.0*	9	*6.3*	14	*10.5*	10	*7.8*	*7.6*	*24.0*	1.36 (0.52–3.54)	0.534	40	30.0 (21.7–40.5)^e^
Any urogenital STI	14	*9.3*	12	*8.4*	6	*4.5*	8	*6.3*	*7.2*	*21.3*	0.62 (0.24–1.61)	0.326	41	30.8 (22.4–41.3)^f^

Bold denotes statistically significant

Italics represent proportions

^a^P value comparing visit 4 vs visit 1, multilevel fixed effects logistic regression, along with the OR and 95% CI

^b^Chlamydia at multiple sites at the same visit counted as one incidence, same for gonorrhoea

^c^Includes incidences of NSU (5), HSV (4), warts (2), proctitis (1)

^d^Includes proctitis and warts; rectal chlamydia and gonorrhoea at same visit counted as two incidences

^e^Pharyngeal chlamydia and gonorrhoea at same visit counted as two incidences

^f^Includes NSU; urogenital chlamydia and gonorrhoea at same visit counted as two incidences

There were no changes in the three monthly incidence of heavy alcohol use before sex, heavy drug use before sex, methamphetamine use before sex, chemsex or drug injection from baseline to visit 4 (Table 2). The overall 12-month incidence of these practices was: heavy alcohol use (17.3%); heavy drug use (10.0%); methamphetamine (8.7%); chemsex (19.3%); injecting drugs (4.0%).

**Fig. 3:**
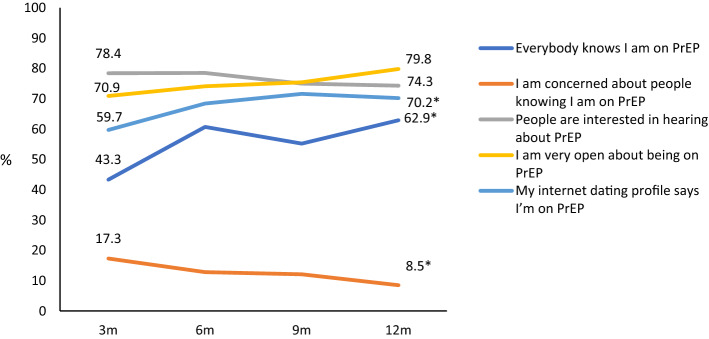
Communication to others about PrEP among NZPrEP participants to 12 months follow-up (*denotes statistically significant trend)

**Fig. 4:**
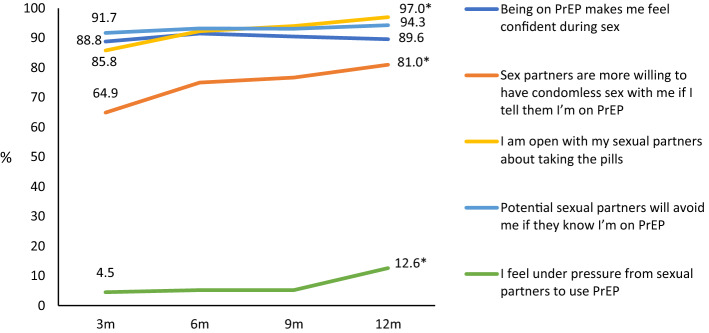
Communication to sexual partners about PrEP among NZPrEP participants to 12 months follow-up (*denotes statistically significant trend)

**Fig. 5:**
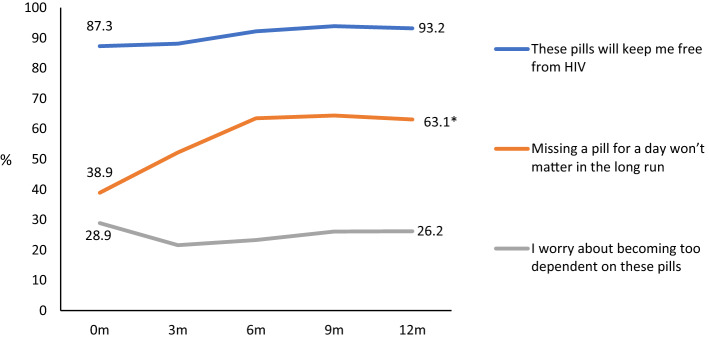
PrEP and the future among NZPrEP participants to 12 months follow-up (*denotes statistically significant trend)

Communication about PrEP increased between visit 1 and visit 4, with more participants agreeing that they mentioned PrEP in their internet dating profile (OR 2.37, 95% CI 1.02–5.50) and that “everybody knows I’m on PrEP” (OR 4.86, 95% CI 2.10–11.28), and a decreasing proportion stating they were concerned about people knowing they were on PrEP (OR 0.25, 95% CI 0.08–0.76) (Fig. 3). Increasingly, participants stated they were open with their sexual partners about taking PrEP (OR 7.41, 95% CI 1.84–29.86) and that sex partners were more willing to have condomless sex if the participant said they were taking PrEP (OR 3.20, 95% CI 1.53–6.69). A small but increasing proportion agreed they felt under pressure from sexual partners to use PrEP (OR 3.96, 95% CI 1.25–12.50) (Fig. 4). Compared to participants’ baseline attitudes, there was a rise in the proportion agreeing that “missing a pill for a day won’t matter in the long run” by visit 4 (OR 4.84, 95% CI 2.33–10.05) (Fig. 5).

### 12 Months Follow up: By Ethnicity

Māori/Pacific participants were as likely to be aged under 30 (45.0% vs 42.7%, chi-square = 0.06, p = 0.805) as non-Māori/Pacific participants. Survey engagement over time was similar for Māori/Pacific and non-Māori/Pacific participants and there was no difference in missed appointments (Table 4).

**Table 4:** PrEP adherence, study engagement, sexual behaviors, STIs and drug use of NZPrEP survey respondents to 12 months follow up, disaggregated by ethnicity

Variable	Baseline		Visit 1 (months 0–3) n = 134		Visit 2 (months 3–6) n = 117		Visit 3 (months 6–9) n = 117		Visit 4 (months 9–12) n = 106		Average per visit	Overall	OR, RR (95%CI)	P value Māori/Pacific vs non-Māori/Pacific
	n	%	n	%	n	%	n	%	n	%	%	%		
Survey engagement														
Māori/Pacific	42	*100.0*	35	*83.3*	33	*78.6*	30	*71.4*	28	*66.7*	n/a	n/a		
Non-Māori/Pacific	108	*100.0*	99	*91.7*	84	*77.8*	87	*80.6*	78	*72.2*				
Missed an appointment														
Māori/Pacific			4	*11.4*	5	*15.2*	5	*17.9*	3	*11.5*	13.9	26.2	1.54 (0.51–4.62)	0.439^a^
Non-Māori/Pacific			12	*12.2*	6	*7.2*	9	*10.3*	9	*11.8*	10.5	21.3		
*PrEP adherence*														
No. missed pills < 30 days; mean (SD)														
Māori/Pacific			2.2	(5.2)	1.8	(2.4)	2.3	(2.9)	5.2	(9.3)	2.8 (5.6)	n/a	**1.84 (1.07–3.18)**	**0.028**^b^
Non-Māori/Pacific			1.3	(1.7)	1.6	(4.1)	1.4	(1.9)	1.9	(4.2)	1.5 (3.1)			
No. missed pills < 3 months; mean (SD)														
Māori/Pacific			4.6	(15)	4.8	(7.6)	5.5	(6)	10.7	(22.9)	6.2 (14.3)	n/a	**2.18 (1.22–3.88)**	**0.008**^b^
Non-Māori/Pacific			2.4	(3.5)	3.2	(10.1)	3.1	(4)	4.1	(10.7)	3.1 (7.6)			
Self-rated “taking the pills is easy” (strongly agree)														
Māori/Pacific			16	*44.4*	14	*42.4*	15	*50.0*	13	*46.4*	45.7	n/a	**0.38 (0.15–0.97)**	**0.043**^a^
Non-Māori/Pacific			58	*58.6*	56	*66.7*	55	*63.2*	46	*59.0*	61.8			
Self-rated “how well did you do at taking the pills?”(extremely well)														
Māori/Pacific			17	*48.6*	20	*60.6*	15	*50.0*	13	*46.4*	51.6	n/a	**0.34 (0.14–0.85)**	**0.022**^a^
Non-Māori/Pacific			66	*66.7*	57	*68.7*	59	*67.8*	54	*69.2*	68.0			
*PrEP breaks and side effects*														
Had a break from pills														
Māori/Pacific			2	*5.7*	3	*9.1*	6	*20.0*	8	*28.6*	15.1	35.7	**4.36 (1.45–13.1)**	**0.009**^a^
Non-Māori/Pacific			3	*3.1*	4	*4.8*	7	*8.1*	7	*9.0*	6.1	15.7		
*Sexual behaviors*														
MenAICLR; mean(SD)														
Māori/Pacific	4.0	(4.1)	2.8	(3.5)	3.3	(3.8)	3.2	(3.9)	3.4	(4.3)	3.4 (3.9)	n/a	**0.65 (0.46–0.91)**	**0.012**^b^
Non-Māori/Pacific	5.2	(6.2)	5.8	(8.6)	6.2	(10.6)	6.0	(10.7)	6.0	(11.6)	5.8 (9.5)			
Any MenAICLR														
Māori or Pacific	38	*95.0*	28	*80.0*	29	*87.9*	23	*82.1*	19	*76.0*	85.1	81.0	0.33 (0.08–1.33)	0.119^a^
Non-Māori/Pacific	103	*97.2*	86	*88.7*	71	*85.5*	82	*94.3*	66	*86.8*	90.9	88.9		
10 or more MenAICLR														
Māori/Pacific	2	*4.8*	1	*2.9*	2	*6.1*	3	*10.7*	3	*12.0*	6.8	n/a	0.25 (0.05–1.22)	0.086^a^
Non-Māori/Pacific	13	*12.0*	17	*17.5*	11	*13.3*	16	*18.4*	11	*14.5*	15.1			
*Sexually transmitted infections*														
Any STI														
Māori/Pacific			12	*28.6*	13	*33.3*	7	*20.0*	9	*28.1*	29.0	64.3	0.93 (0.54–1.59)	0.781^a^
Non-Māori/Pacific			38	*35.2*	27	*26.0*	32	*32.3*	21	*21.9*	27.7	63.9		
Rectal STI														
Māori/Pacific			7	*16.7*	4	*10.3*	5	*14.3*	3	*9.4*	14.8	31.0	**0.42 (0.19–0.94)**	**0.034**^a^
Non-Māori/Pacific			29	*26.9*	19	*18.3*	28	*28.3*	15	*15.6*	22.3	50.0		
*Substance use*														
Alcohol use before sex < 3 m (heavy)														
Māori/Pacific	8	*19.1*	6	*17.1*	3	*9.1*	4	*14.3*	4	*15.4*	15.2	21.4	2.0 (0.39–10.23)	0.407^a^
Non-Māori/Pacific	12	*11.1*	9	*9.1*	9	*10.8*	12	*13.8*	7	*9.1*	10.8	15.7		
Drug use before sex < 3 m (heavy)														
Māori/Pacific	5	*11.9*	3	*8.6*	3	*9.4*	2	*7.1*	0	*0.0*	8.0	9.5	1.31 (0.19–9.02)	0.785^a^
Non-Māori/Pacific	10	*9.4*	4	*4.0*	4	*4.8*	4	*4.6*	3	*3.9*	5.5	10.2		
Methamphetamine use before sex < 3 m (any)														
Māori/Pacific	3	*7.1*	2	*5.7*	1	*3.1*	1	*3.6*	0	*0.0*	4.3	9.5	0.65 (0.06–7.46)	0.730^a^
Non-Māori/Pacific	9	*8.4*	7	*7.1*	5	*6.0*	4	*4.6*	4	*5.2*	6.4	8.3		

*n/a* not applicable, *SD* standard deviation

Bold denotes statistically significant

Italics represent proportions

Overall cumulative % may be lower than a visit % for some outcomes due to survey attrition. MenAICLR = No. male partners had condomless receptive anal intercourse with

P values compare outcome for Māori/Pacific vs non-Māori/Pacific after adjusting for study visit, along with the OR or RR and 95% CI, using the following statistical tests:

^a^For categorical data, multilevel fixed effects logistic regression

^b^For continuous data (counts), multilevel fixed effects Poisson regression

After adjusting for study visit, Māori/Pacific participants were more likely than non-Māori/Pacific participants to report missing four or more PrEP doses in the last 30 days prior to visit (19.1% vs 8.9%; OR 4.55, 95% CI 1.18–18.57) and reported a higher number of missed pills over the last 30 days and the three months prior to visit. Māori/Pacific participants were less likely than non-Māori/Pacific participants to strongly agree that “taking the pills is easy” (45.7% vs 61.8%; OR 0.38, 95% CI 0.15–0.97) and to state they did “extremely well” at taking the pills (51.6% vs 68.0%; OR 0.34, 95% CI 0.14–0.85) (Table 4).

Māori/Pacific participants were significantly more likely than non-Māori/Pacific participants to report taking a PrEP break (Table 4). The average across visits was 15.6% vs 6.1% (OR 4.36, 95% CI 1.45–13.1) and by visit 4 it was 28.6% vs 9.0%; overall 35.7% of Māori/Pacific participants took a break at least once compared to 15.7% of non-Māori/Pacific participants. There were no differences between ethnicity groups in the incidence or severity of side-effects (not shown).

Māori/Pacific participants generally reported fewer male sexual partners compared to non-Māori/Pacific participants (Table 4). For example, Māori/Pacific participants reported fewer condomless receptive anal intercourse partners compared to non-Māori/Pacific participants in the three months prior to visit. There were no differences in the incidence of clinically-diagnosed chlamydia, gonorrhoea or syphilis for Māori/Pacific participants (data not shown) nor in the combined “any STI”. However Māori/Pacific participants were less likely to be diagnosed with a rectal bacterial STI (overall diagnoses 31.0% vs 50.0%; OR 0.42, 95% CI 0.19–0.94) (Table 4). No significant differences were found between Māori/Pacific and non-Māori/Pacific participants in the incidence of heavy alcohol or drug use before sex or methamphetamine use before sex (Table 4).

## Discussion

We believe this prospective cohort study of GBM in Auckland, New Zealand is the first to examine PrEP use by ethnic group outside the US. Overall, we found high PrEP adherence, no increases in condomless sex, stable although high STI incidence and improving communication about PrEP with others. However, Māori and Pacific participants exited the study earlier, were less adherent to PrEP, found taking PrEP more difficult and were significantly more likely to take PrEP breaks, compared to non-Māori/Pacific participants. Māori/Pacific GBM on PrEP reported fewer condomless sexual partners but a similar STI incidence to non-Maōri/Pacific GBM, indicating potential for HIV infection should PrEP be interrupted. Our study adds important international data implying inherent health system-related disparities for non-white GBM taking PrEP. Unless these are addressed, current modes of PrEP delivery have the potential to widen HIV inequities for ethnic minority GBM.

New Zealand offers an important perspective on PrEP implementation for several reasons. Published accounts of “gold-standard” PrEP delivery often arise from cities with concentrated urban gay communities like Sydney, London or Amsterdam [68] with highly developed sexual health infrastructure positioned close to gay precincts. In contrast to these outliers, Auckland has a geographically dispersed population with high ethnic diversity. With only four publicly funded sexual health clinics servicing the city, each with limited capacity to prioritise GBM alongside other vulnerable heterosexual populations, it lacks such GBM-focused sexual health infrastructure [56]. For these reasons, Auckland potentially better reflects the typical PrEP delivery landscape that most GBM internationally must navigate. Our study also coincides with a “switchover” moment in HIV prevention delivery from community-based to clinic-based biomedical approaches [69]. This allows us to explore the benefits of PrEP, but also its risks, including the potential over-medicalisation of gay men’s sexual lives and increasing gay men’s dependence on heterosexist and racist health systems [56]. Other strengths include the study using the publicly-funded PrEP criteria, the prospective cohort design, clinically-validated STI diagnoses, the linked anonymous survey participation and the wide range of variables collected.

Limitations of our study include the small sample size, the self-reported behavioral data that may be subject to recall bias and cannot be validated and incomplete surveys. Early PrEP adopters may not be representative of all GBM now eligible for publicly funded PrEP in New Zealand [57]. We present various measures of sub-optimal PrEP adherence, but most participants were taking PrEP at protective levels when not intentionally on a break. Combining Māori with Pacific ethnicity increased study power, however each will have distinct experiences and this is not a preferred approach. We did not have study power to examine ethnic minority Asian GBM, who exhibit distinct PrEP experiences in New Zealand [70], nor migrant GBM who do not qualify for funded PrEP. Multiple statistical tests were not adjusted for in this study, which runs a risk of type 1 errors. Our findings may not reflect the experience of accessing PrEP in primary care, nor event-based PrEP dosing (“2-1-1”).

Our finding of disparities in PrEP experiences for Māori and Pacific GBM support research from the US indicating lower PrEP awareness, lower retention in real-world PrEP studies, lower PrEP adherence and lower clinic engagement among Black GBM [28, 34, 36–38, 40, 48, 49, 51–53, 71]. As with other infectious diseases like COVID-19 [72], poorer outcomes for HIV PrEP among Black GBM in the US are influenced not only by individual and motivational factors, but also health system factors such as clinician bias and stigma, and structural factors such as poverty, housing and mobility [53, 54, 73]. Similar structural factors are likely to trouble PrEP engagement for Māori/Pacific GBM in New Zealand.

In our baseline study, Māori and Pacific GBM were just as likely to have volunteered for PrEP as non-Māori/Pacific GBM, and there were no differences in risk practices on entry. Instead, disparities emerged post-enrolment. Compliance with our study’s PrEP programme required time off work, travel across a geographically dispersed city, sufficient privacy to take medications and complete regular online surveys, and social and peer support to continue PrEP over 12 months, conditions which could impose additional barriers for Māori and Pacific GBM due to colonisation. To ensure the benefits of PrEP are equally enjoyed by ethnic minority GBM, health systems must identify and eliminate institutional racism, and offer minorities sexual health services that respond both to their HIV risk but also to the lived experiences of ethnic minority GBM [4, 47, 73, 74].

No participant acquired HIV during the study, although the high STI incidence is consistent with international literature [27, 43–45]. Among our cohort, STI prevalence was high on entry; the three-month and 12 month incidence of rectal bacterial STIs confirms these GBM were at high ongoing risk of acquiring HIV in absence of PrEP. Gonorrhoea and syphilis cases were rising among New Zealand GBM prior to PrEP [75]; for our elevated-risk subset, the three-monthly clinic visits likely reduced undiagnosed infections and aided control.

Endemic STIs and frequent STI screening among GBM taking PrEP also have attendant public health implications. Sexual health clinics in this study struggled with capacity and continue to do so, as demand for PrEP has increased following public funding alongside an outbreak of syphilis (including congenital cases). Planners and funders should resize services to match increased demand, ensure the sexual health needs of other priority groups are met, and seek innovative PrEP delivery mechanisms to overcome implementation challenges [56]. We know little about STI incidence, testing and treatment in GBM who are “PrEP adjacent” i.e. not on PrEP but sexually active with GBM who are. Many of those GBM may not wish to engage routinely with clinical services, or have access to them locally. Consequently, we believe gay men’s community agencies should continue promoting condoms and home testing [76] as an effective intervention for both HIV and STIs.

The most noticeable changes over 12 months were attitudes to PrEP. Participants became more likely over time to tell others they were on PrEP, including sexual partners, and were less concerned about people knowing. This could reflect diminishing PrEP stigma among friends, family and other GBM once it was publicly funded in New Zealand and received mainstream media coverage. A number of studies show that PrEP stigma negatively impacts PrEP users and PrEP-interested GBM [77], increasing the likelihood of medication concealment, poorer adherence, discontinuation or PrEP-seeking. Re-framing PrEP use as a way of taking responsibility for one’s sexual health or reducing HIV anxiety could help further erode PrEP stigma, both within GBM communities and public health circles [77, 78]. Open disclosure of PrEP status appeared to benefit participants: an increasing proportion stated that sex partners were more willing to have condomless sex if they did so. At the same time, a smaller proportion reported they felt under pressure from sexual partners to use PrEP. As Australian researchers have shown, high uptake of PrEP among Sydney gay men in 2015–2018 was followed by increasing stigma directed at non-PrEP approaches to safer sex, introducing new community divisions [79]. The ongoing diversification of safe sex tools for HIV and STIs has implications for health promotion among GBM. For example, community organisations can help GBM weigh the pros and cons of different risk reduction options and encourage GBM to respect the choices their sexual partners make.

Future PrEP cohorts should consider equity quotas so they are sufficiently powered to examine ethnic minority experiences. Administrative data such as funded PrEP prescriptions would provide intelligence on disparities in uptake and continuation. Community behavioral surveillance should examine PrEP awareness, willingness, eligibility and uptake [80], and interest in long-acting or on-demand PrEP options for ethnic minority GBM that could minimize barriers to drug adherence and clinic engagement [81]. Findings should be triangulated to describe the “PrEP cascade” for GBM and whether this varies by ethnicity.

## Conclusions

In Auckland, New Zealand, Māori/Pacific GBM had a similar demographic and behavioral profile on study entry but disparities emerged after taking PrEP. Eliminating disparities in the PrEP journey for Māori/Pacific GBM will likely require interventions that target individual, service delivery and structural factors. This is critical since clinic-based interventions such as PrEP are becoming the dominant HIV prevention approach for GBM internationally.

## Supplementary Information

Below is the link to the electronic supplementary material.

Supplementary file1 (DOCX 16 KB)

## Data Availability

Data access requests should be submitted to the corresponding author.
