# Supplementary material for: Adherence, Sexual Behavior and Sexually Transmitted Infections in a New Zealand Prospective PrEP Cohort: 12 Months Follow-up and Ethnic Disparities
Source: AIDS Behav. 2022 Feb 15;26(8):2723–37. doi: 10.1007/s10461-022-03617-5 (PMC8853116; doi:10.1007/s10461-022-03617-5)
Supplement: Supplementary file 1 — Supplementary file1 (DOCX 16 KB) [file 10461_2022_3617_MOESM1_ESM.docx]

**SUPPLEMENTARY MATERIAL**

**Supplementary Table 1** Reasons why participants missed PrEP doses (n=326 missed pill disclosures)

| Reason | n | % |
| --- | --- | --- |
| I forgot | 166 | 50.9 |
| I was away from home | 145 | 44.5 |
| My daily routine changed | 111 | 34.1 |
| I was too busy | 35 | 10.7 |
| I was tired | 25 | 7.7 |
| I ran out of pills | 21 | 6.4 |
| I was not feeling well | 14 | 4.3 |
| I was stressed or anxious | 8 | 2.5 |
| I was taking recreational drugs | 7 | 2.2 |
| I was worried about interactions with other drugs | 6 | 1.8 |
| I had side effects from taking the pills | 6 | 1.8 |
| I did not want people to see me taking pills | 2 | 0.6 |
| I lost my pills | 2 | 0.6 |
| I thought it would make me feel sick | 2 | 0.6 |

**Supplementary Table 2** Reasons why participants took a break from PrEP (n=40 break disclosures)

| **Reason** | **n** | **%** |
| --- | --- | --- |
| I was not having sex | 12 | 30.0 |
| I didn’t feel at risk of HIV | 7 | 17.5 |
| I was worried about interactions with other drugs | 7 | 17.5 |
| I was doing event-based dosing | 6 | 15.0 |
| I was using condoms | 5 | 12.5 |
| I was not feeling well | 4 | 10.0 |
| I wanted to save the pills for another time | 3 | 7.5 |
| I had trouble getting a refill | 3 | 7.5 |
| I ran out of pills | 2 | 5.0 |
| I was confident my HIV positive sex partner was undetectable | 2 | 5.0 |
| I gave my pills to someone else | 1 | 2.5 |
| I had side effects form the pills | 1 | 2.5 |
| I couldn’t be bothered | 1 | 2.5 |
| I was doing intermittent dosing | 0 | 0.0 |
| I was confident my sex partner was not HIV positive | 0 | 0.0 |
| I lost my pills | 0 | 0.0 |
| I was using recreational drugs | 0 | 0.0 |

**Supplementary Fig. 1** Study retention over 12 months follow-up by ethnicity group
